# A passive positioning alarm used by persons with dementia and their spouses – a qualitative intervention study

**DOI:** 10.1186/1471-2318-13-11

**Published:** 2013-02-05

**Authors:** Annakarin Olsson, Maria Engström, Claudia Lampic, Kirsti Skovdahl

**Affiliations:** 1 Faculty of Health and Occupational Studies, University of Gävle, Gävle, 80176, Sweden; 2 School of Health and Medical Sciences, Örebro University, Örebro, 701 82, Sweden; 3 Department of Neurobiology, Care Sciences and Society, Karolinska Institutet, Stockholm, 171 77, Sweden; 4 Department of Public Health and Caring Sciences, Uppsala University, Uppsala, Sweden; 5 Faculty of Health Sciences, Buskerud University College, Drammen, Norway

**Keywords:** Alzheimer´s disease, Information and communication technology (ICT), Interview, Participant observation, Passive positioning alarm, Spouse, Tracking

## Abstract

**Background:**

Increasingly, information and communication technology is being used to support persons with dementia living at home and their relatives. The aim of the present intervention study was to describe and explore the use and experiences of using a passive positioning alarm, over time, in daily life among persons with dementia and their spouses.

**Methods:**

Using an ethnographically inspired approach, five couples, each including a person with Alzheimer´ s disease and his/her spouse living in their own home, were repeatedly observed and interviewed regarding their experiences of using a passive positioning alarm. Interview text transcripts and field notes were analyzed using qualitative content analysis.

**Results:**

The main findings show changes over time, where testing and checking the passive positioning alarm successively led to trust in the alarm and in one own´s ability to use it. These components were a prerequisite for the couples to perceive the value of the alarm.

**Conclusions:**

A passive positioning alarm for persons with dementia and their spouses needs to be packaged as a “service” with flexibility for each user and based on user needs, abilities, knowledge and skills. Using a passive positioning alarm can be a valuable support that allows persons with dementia to be alone outdoors and can increase safety and security for them and their spouses. The present study contributes to our understanding of what prerequisites need to be in place and what barriers need to be dealt with before successful implementation can occur.

## Background

Being outdoors on one’s own is considered an important activity of daily living and, for many; it is associated with the ability to maintain independence. A dementia diagnosis is distressing in its own right, but when paired with the threat of losing one’s independence, the issue of the living situation becomes problematic for both the person with dementia and his/her relatives [1]. One way to help persons with dementia be outdoors by themselves and to increase safety and security for relatives and persons with dementia may be to use different kinds of information and communication technologies (ICT) [2].

Living with a progressive disease such as dementia affects the entire life situation [3], not only for the person with dementia, but also for his/her relatives [4,5]. For persons with dementia, managing daily life often requires the help and support of relatives [6]. Several studies have shown that relatives of a person with dementia experience a feeling of being burdened [1,7-9]. The impairments accompanying the disease, e.g., memory and orientation difficulties, may lead to safety issues, such as the person with dementia getting lost [10], and may even force relatives to lock exterior doors or in other ways prevent the person with dementia from going outdoors [11,12]. Being outdoors is seen by healthy persons as well as persons with dementia as something valuable and important [13] and studies have revealed that being in nature environments has health benefits [14,15]. Thus, it would seem to be important to develop technologies that make it easier for persons with dementia to be outdoors on their own in a way that allows them and their relatives to feel safe and secure.

According to research in the area, use of ICT in the care of persons with dementia has increased [2,16,17]. A wide range of technologies exist that are intended to support the daily life of persons with dementia and their relatives, e.g., GPS (global positioning system) tracking technologies [18-20]. GPS provides a means of locating the user at any given moment by locating the device via satellite and sending the information via a network to a personal computer, a call center or a cell phone [21]. A review by Lauriks and colleagues [2] showed that tracking technologies promote perceived safety and security among relatives of persons with dementia in that such devices allow relatives to locate the person if he/she is lost. Recently published studies by Olsson et al. [22] and White et al. [20] have revealed that relatives of persons with dementia perceived ICT as a support in everyday life. However, earlier studies in this area have primarily taken the perspective of relatives and/or healthcare staff, and the need to investigate the perspective of persons with dementia has been emphasized [16,23-25]. Studies including the perspective of persons with dementia by interviewing them have mostly concerned ICT other than tracking technologies (e.g. [12,26]), and few of these have involved persons with dementia still living in their own homes (e.g. [19,27]. Some observational studies concerning ICT use among persons with dementia have been identified [28,29], however these studies did not focus on GPS tracking. Using tracking technologies in the care of persons with dementia has also raised ethical concerns concerning, e.g., the risk of violating the privacy and dignity of these persons [24,30-32], and critical voices have also been heard in society at large suggesting that the technology could be seen as a form of surveillance [30]. As such, it is important to further investigate users’ use and experiences of ICT in everyday life, both persons with dementia and their relatives.

Only a limited numbers of studies [18,24] have explored and/or described experiences of using GPS tracking technology from the perspective of persons with dementia. Observational studies on how involved actors use as well as relate to GPS tracking are important if we are to create need-driven and individually adapted technology. The aim of the present intervention study was to describe and explore the use and experiences of using a passive positioning alarm, over time, in daily life among persons with dementia and their spouses.

## Methods

### Design

A qualitative intervention study with a descriptive and explorative design was used to investigate variation across individuals and evaluate the intervention in their real-life contexts [33].

### Participants and setting

The study was performed in one municipality in central Sweden, from October 2010 to March 2011. Inclusion criteria were: couples consisting of a person diagnosed with a dementia disease and his/her relative cohabiting in their own home, the person with dementia should have a desire and need to be outdoors, and both partners should be able to communicate verbally in Swedish. A purposive sample [34] of five couples, including persons with dementia varying in age and sex, was recruited by healthcare staff at the memory unit within the county council district (n=1) and the Relative Caregivers Support Centre (n=4). Prior to study participation, all couples had experienced incidents in which the person with dementia had had difficulties finding his/her way home while alone outdoors. Fear of the person with dementia getting lost was expressed by both the persons with dementia and their spouses (Table 1).

**Table 1:** Demographics of the persons with dementia and spouses

	**All**	**Women**	**Men**
**Persons with dementia**			
Sex	5	2	3
Age (Md years)(range)	68(55–73)	60	71
Years since diagnosis (Md time)	6	6	6
MMSE* (Md)(range)	23(19–28)		
**Spouses**			
Sex	5	3	2
Age (Md years) (range)	67 (62–68)	67	65
**Living**			
Terrace house	3		
Detached house	1		
Apartment	1		
Summer cottage	2		
**Daytime activity program**			
Frequency (day-s/week)**	3	2	1

* MMSE= Mini Mental State Examination (valued measured < 1 year ago).

** Varied between 1–3 days/week.

The persons with dementia were all diagnosed with Alzheimer´ s disease at an early (n=4) or middle (n=1) stage of dementia (ICD-10). All couples were married and lived outside a city. All persons with dementia performed daily outdoor activities, predominantly alone, including taking short walks in the neighborhood or longer walks in the forest.

### The passive positioning alarm package (PPAP) intervention

**Figure 1:**
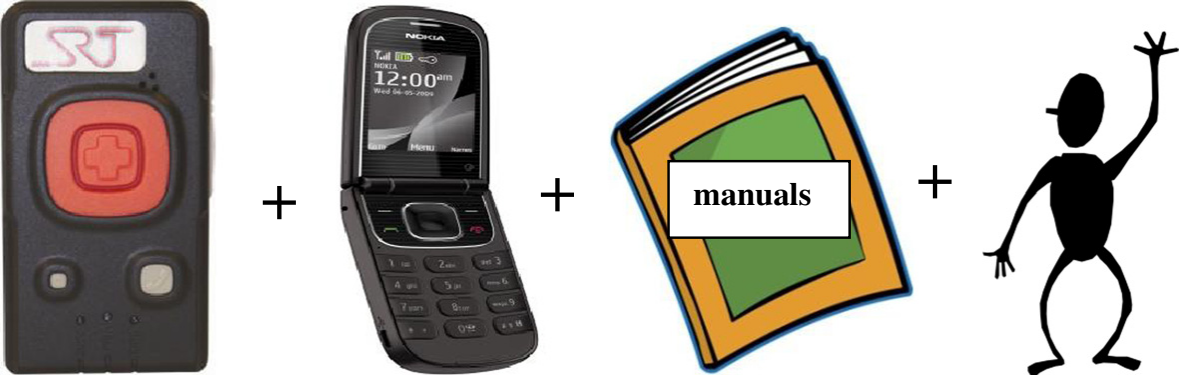
“**PPAP**” – **transmitter**, **cell phone**, **manuals and support person.**

The intervention comprised a “service package” containing a transmitter (based on GPS technique), a cell phone, manuals for the transmitter and the cell phone and a support person (who demonstrated the passive positioning alarm and could be reached by telephone) (Figure 1). When the persons with dementia went on their daily outdoor walks, they carried the transmitter in their pocket, glove or bag. Before the person with dementia left the home (or other place), the transmitter was activated by the spouse, who pushed a big red button marked with a cross, thereby creating a virtual fence with a radius of 500 meters. When the person with dementia left this predefined area, a Short Message Service [SMS] with a map was sent to the spouse´s cell phone, enabling the spouse to see the location of the person with dementia. Before the present study began, the passive positioning alarm package (PPAP) was tested for a period of six weeks by healthy elderly persons without known cognitive impairments.

Two weeks before the start of data collection, the couples received their transmitter, manuals for the transmitter and cell phone along with a two-hour verbal instruction session provide by the support person involved in the project. The support person was a healthcare professional from the municipality with long experience of working with persons with dementia. During the instruction session, the couples were able to test the transmitter and cell phone, ask questions and read the manuals. The support person was responsible for providing technical support throughout the study period.

### Procedure

#### Participant observations and interviews

Inspired by an ethnographic approach [35] repeated participant observations and interviews were used to describe and explore the couples’ experiences and to describe how they used and handled the PPAP. The observations were conducted in the couples´ homes and during outdoor walks with the persons with dementia. For each couple, data collection was performed on five occasions (seven occasions for one couple). The observations focused on the couples´ handling of the transmitter and cell phone. The interviews, henceforth called informal conversations [34], focused on how the persons with dementia and their spouses experienced using the PPAP. A co-observer (another healthcare professional with long experience of working with persons with dementia, not the support person) participated in 17 of the 27 data collection occasions [34]. Use of a co-observer allowed one observer to accompany and observe the person with dementia on his/her outdoor walk, while the other observer observed and had an informal conversation with the spouse. The researcher and the co-observer alternated roles during the data collection occasions. On all data collection occasions, a summary was made (together when there were two observers present) immediately after the data collection, where field notes were written down and data were synchronized [34].

The first and last authors conducted the first two data collection occasions together, the goal being to develop ideas about how to best carry out the observations and informal conversations. Details and impressions were discussed between the researchers after the initial data collection occasions, concerning, e.g., the appropriate (physical) location from which to visually obtain the most information before and after the person with dementia took his/her outdoor walk. For all couples, the first data collection occasion occurred two weeks after they had received the PPAP. On this data collection occasion, the persons with dementia and their spouses were encouraged to describe their everyday life (focusing on the outdoor activities of persons with dementia) and to describe why they were interested in participation in the present study focusing on use of a passive positioning alarm. The first occasion also seemed to be important in creating confidence and a trusting relationship.

**Figure 2:**
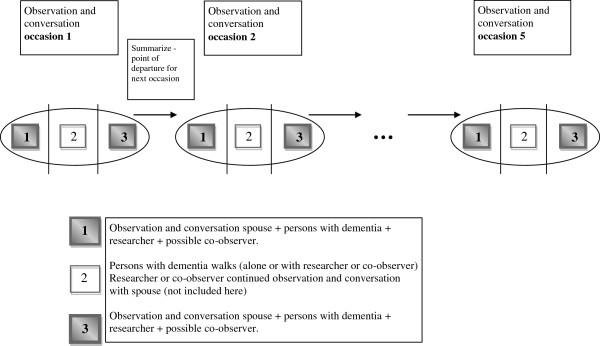
Data collection process.

All subsequent data collection occasions followed a specific pattern (Figure 2). First a joint informal conversation with the person with dementia and his/her spouse took place, where the couple summarized what had happen in their daily life, in relation to use of the PPAP, since the previous data collection occasion (Part 1). Then the person with dementia went for an outdoor walk, during which he/she was encouraged and instructed to walk as he/she normally did when alone. During this walk, an informal conversation was conducted with the person with dementia and simultaneously, an informal conversation was conducted with the spouse in the home (Part 2). Finally, when the person with dementia returned home, a joint informal conversation was held with the person with dementia and his/her spouse, focusing on the former´s experiences of the outdoor walk, in relation to use of the PPAP (Part 3). To facilitate and stimulate recall [35,36] of the outdoor walks and to see on the map where the person with dementia had walked, the transmitter and cell phone were placed on the table during this part of the data collection. The couple’s own spontaneous experiences and reflections were then followed up on the subsequent data collection occasions. The time intervals between data collection occasions varied across couples depending on their vacations or other planned activities.

The observations were mostly recorded using an Mp3 player, though sometimes written observations were made. The audio-recorded observations were used to avoid the couples feeling ”studied” and hearing what was recorded. The observer described verbally on the Mp3, e.g., the preparations made (spouses pushing the transmitter button, giving the transmitter to the person with dementia or putting it in his/her pocket, bag or glove) before the person with dementia went on his/her outdoor walk. To maintain complete focus during the observations, most field notes were written immediately after each data collection. The field notes consisted of reflections made during the observations and informal conversations: placement during the observation and informal conversation, physical position of the observer when the person with dementia left the house, perceived state of mind of the spouse and the person with dementia, facial expressions of the spouse and person with dementia, and the weather at the time [34].

### Data analysis

The Mp3 files containing the recorded observations and informal conversations were transcribed verbatim. The transcripts, together with the field notes, were analyzed using qualitative content analysis as described by Graneheim and Lundman [37], Krippendorff [38] and Patton [34]. The field notes provided necessary and important information, allowing the researchers to put the data in the “right” context. First, all transcripts and field notes were read through several times to gain an overview and general impression of the data. Second, meaning units related to the study aim were identified, thereafter condensed and labeled with a code. These steps were performed by the first author and discussed with the last author. Codes that expressed related meanings were grouped together into five sub-categories and two categories. The identified codes, sub-categories and categories were discussed in the entire research team. Analysis and findings from observations and informal conversations during the outdoor walks (Part 2), concerning the persons with dementia and their strategies for and feelings about being alone outdoors, will be reported elsewhere (Figure 2).

### Ethical considerations

Approval was obtained from The Regional Ethical Review Board (2009/078). The participants received oral and written information about the study and both the person with dementia and the spouse gave their written informed consent. Participation in the study was strictly voluntary and all participants were assured of confidentiality. No participant declined participation. Furthermore, methodological and ethical considerations especially relevant in research on persons with dementia were taken into account [39], such as the fact that observations and conversations might be emotionally stressful and raise feelings of discomfort among both persons with dementia and relatives. It was therefore important for the researcher to approach the situation with sensitivity, to look for signs of distress and, if necessary, to end the observation and/or conversation.

## Results

**Figure 3:**
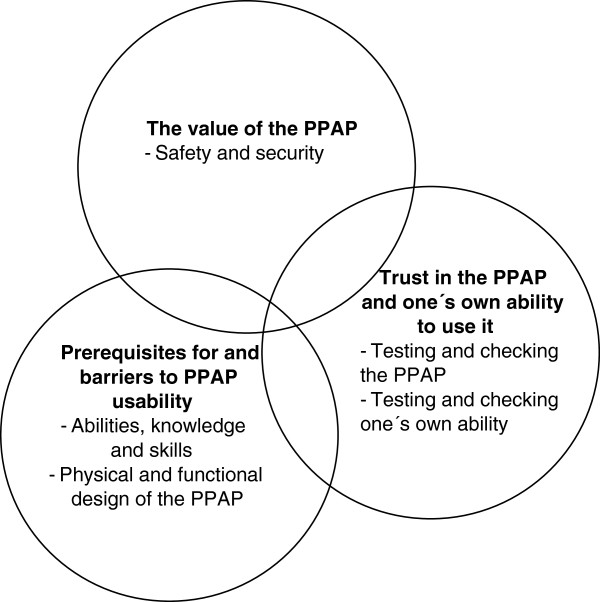
**Categories and sub**-**categories describing the results.**

The participants’ experiences of the PPAP appeared to change over the time in which the couples were observed using and becoming familiar with the PPAP. The progression toward comfortable and competent use and handling of the PPAP was individual, owing to crucial factors such as abilities, knowledge and skills in using the device. By testing and checking the PPAP and their own performance, they developed a trust in the PPAP and in their own ability to use it. In parallel with this increased trust, they also described the PPAP as valuable. The findings are presented in three categories and four sub-categories (see Figure 3); the categories “Prerequisites for and barriers to PPAP usability” and “Trust in the PPAP and one’s own ability to use it” are considered preconditions for the category “Value of the PPAP”.

### Prerequisites for and barriers to PPAP usability

The couples described and showed the abilities, knowledge and skills needed to use and handle the PPAP. The design aspects of the PPAP that needed to be revised, changed and/or complemented were discussed by both the spouses and the persons with dementia.

#### Abilities, knowledge and skills

The spouses’ described their ICT abilities, knowledge and skills as varying from limited to more extensive. The spouses who described previously or still working with ICT reported being and were shown to be more comfortable with using the PPAP. In some couples, the person with dementia was primarily the one who had handled, or was still handling, technical devices (e.g., remote controls, DVD player, etc.) in the home, and in other couples the spouse was the more technical person. Those who felt they had and showed limited abilities, knowledge and skills reported on the first data collection occasion that they needed further verbal and hands-on instructions from a support person if they were to use the transmitter and cell phone and understand the manuals. They showed and expressed problems with, e.g., picking up an SMS with the map, reading and understanding the manuals (instructions), getting the transmitter and cell phone started or just knowing and understanding whether the transmitter and cell phone batteries were fully charged. As some spouses said: ‘*Then it depends on how technical you are and I*’*m definitely not*, *right*? *How familiar you are with cell phones in general*’ [spouse occasion 1] and `*I*´ *m not so great at technical things so I think*, *can I do this*?´ [spouse occasion 2]. Spouses who described themselves as more skilled ICT users expressed and showed confidence in handling the technology across all the repeated data collection occasions.

#### Physical and functional design of the PPAP

Both the persons with dementia and their spouses discussed the need for improvements and changes in the design of the whole service package, irrespective of previous abilities, knowledge and skills in relation to different kinds of ICT. Some persons with dementia made statements about desired changes in the design of the PPAP during the first data collection occasion, while others never mentioned changes or described them later on. Among the spouses, all made statements about the need for design changes, but like the persons with dementia, these statements came at different times throughout the data collection. One spouse rewrote the manuals after having seen the transmitter and phone demonstration, `*There’s too much information, reduce the number of steps´* [spouse occasion 1] and `*step-wise instructions would be desirable´* [spouse occasion 1]. Furthermore, the persons with dementia said that in situations when they felt lost outdoors, it was important to be able to contact and hear/talk to their spouses. The spouses also felt this was important, *`the telephone button should be activated (work)…// so we can communicate with each other´* [transmitter] [spouse and person with dementia occasion 2]. Based on their previous ICT abilities, knowledge and skills and their need for increased control, one couple manipulated the PPAP by not activating a new virtual fence when the person with dementia went alone outdoors. Thanks to this manipulation, the position of the person with dementia was constantly visible to the spouse. Other couples also expressed wanting this function, because they felt it would make them feel both safe and secure, *`would like a non-hidden safety zone…//I should always be visible´* [cell phone] [person with dementia occasion 5].

### Trust in the PPAP and one’s own abilities

During the entire data collection, the spouses expressed and showed a need to test and check the PPAP, and they sought verification from the researcher regarding their use and handling of the ICT. The tests and checks were made on the basis of the couple’s abilities, knowledge and skills in using the PPAP. Whether or not they felt they could depend on the PPAP was based on their trust in the PPAP and in their own ability to use and handle it.

#### Testing and checking the PPAP

The spouses reported frequent testing and checking of the PPAP. To do this, the couples took joint walks outdoors and constantly followed their own path on the cell phone. *`Now we´ ve gone out together the last time taking it *[transmitter] *with us just to see if it worked´* [spouse occasion 1]. *`Then I took both the phone and the GPS* [transmitter] *with me when I walked in the woods’* [spouse occasion 3]. This approach was described by the male spouses. The spouses also checked by asking the person with dementia, when he/she returned from the outdoor walk alone, whether he/she could describe (remember) where he/she had walked or they gave the person with dementia a cell phone, called him/her and checked the position on the cell phone. This was both described by the spouses and observed by the researcher.

#### Testing and checking one´ s own ability

To determine whether they were handling the PPAP “correctly,” the spouses showed the researcher what testing and checking one’s own ability could entail, e.g., the different steps in navigating and pushing buttons on the PPAP and cell phone and picking up the map (SMS) as a way of verifying that they were using the ICT in the “right way”. This approach (testing and checking one´ s own ability) was observed more frequently among the female spouses than among the male spouses. As one spouse described: `*When it says “go to” then I know exactly what I should do. But it often shows… sometimes it shows the stock-exchange rates. So should I push the one down here* [cell phone] *because then I’ll get a (signal)… now it’s just a mess… I’m… I doing it wrong now? Because I’m not getting a map. Wait a minute, now I forgot… how can I see the map now? Now there’s a connection. Now I’m supposed to push it* [transmitter] *here right. Right, wasn’t that it?* [spouse occasion 2].

### The value of the PPAP

Both the persons with dementia and their spouses expressed, at different points in time, the value of the PPAP in their life. In parallel with increasing trust in the PPAP and in their own ability to use and handle it, they expressed the value of the PPAP. But the value of the alarm was also described with some caution, as one spouse said: *`I think it´ s good. As long as I make sure she has it* [transmitter] *with her, in her pocket when she leaves… // I try to do this´* [remember]. `*Then I usually put it* [transmitter] *on the chest of drawers there so… she knows she´ s supposed to take it with her´* [spouse occasion 3]. The reported need for and value of the PPAP differed both between and within couples.

#### Safety and security

The persons with dementia appreciated the PPAP as a daily support in compensating for potential physical limitations, e.g. fear of falling while alone outdoors, but indicated that they could not see the PPAP as providing support for their dementia disease today. Some persons with dementia described perceptual impairment as a consequence of disease progression, while others expressed little or no effect of their dementia disease and limitations related to it. The persons with dementia reported being aware that they would one day be much worse and have a greater need for the PPAP, not just for their own safety and security but also for their spouse’s feelings of safety and security. Most of the spouses felt that the person with dementia had some cognitive problems and the spouses expressed a desire and/or a need for the PPAP to support daily outdoor life for the person with dementia. These needs were based on incidents in which the person with dementia could not find his/her way back home while being outdoors on his/her own or had repeatedly absconded from home. One spouse described it as follows: *`I don´ t know if she´ d *[person with dementia] *dare… I don´ t know, it could happen … // so really you need one* [transmitter] *that she wears. Pretty much all the time… // that she wears all the time, then I wouldn´t need to worry about her going out, then I´ d find her´* [spouse occasion 3]. The value of the PPAP was also confirmed by the fact that all couples wished to keep it after the study was completed. One spouse expressed it like this: `I would like to keep it [the passive positioning alarm] …// because it really provides security´ [spouse occasion 5]. The couples also said that the PPAP would be an important safety and security aid for them when spending time both in their summer cottages and in an unfamiliar environment. One person with dementia described how the PPAP could help her move in the area around her cottage, e.g. picking berries and mushrooms, without being afraid of not finding the way home, *`I don´t dare go to places I don´t recognize, so I avoid it´* [person with dementia occasion 3].

The persons with dementia and their spouses saw the value of being locatable and saw no problem with the persons with dementia being monitored; they had not even considered that aspect. One person with dementia said: *`I like the idea that I put it* [transmitter] *on and then he* [spouse] *knows where I am´* [person with dementia occasion 3]. In response to the researcher’s question concerning possible feelings of being monitored, both the persons with dementia and the spouses said that being visible on the map outweighs the risk of having their privacy violated, or as one person with dementia expressed it: *`But that’s what we *[persons with dementia] *want, to be seen!´* [person with dementia occasion 3]. The possibility for spouses to use their cell phone to follow the person with dementia on his/her outdoor walks alone was described as creating both a feeling of safety and security for the couple and the possibility of freedom for the person with dementia. One person with dementia said: *`Now when there´ s so much talk about being locked in, dementia and all… then you can´ t see it as a restriction, you have to turn it around and see it as a possibility´* [person with dementia occasion 5].

## Discussion

The aim of the present intervention study was to describe and explore the use and experiences of using a passive positioning alarm, over time, in daily life among persons with dementia and their spouses. The main findings revealed changes over time in the participants use and experiences of the PPAP, such that testing and checking the PPAP successively led to trust in the PPAP and in one´ s own ability to use it. Testing and checking seemed to be a prerequisite for the couples to perceive the value of the PPAP in their everyday outdoor life. However, individual variations were observed, within and between couples, based on conditions for learning to use, using and handling the PPAP.

The usability of the PPAP was expressed by the couples in terms of the value of the PPAP. The persons with dementia and their spouses described a feeling of safety and security related to use of the PPAP, that is, they perceived it as valuable in situations that were important to them, e.g., being able to move around freely at the cottage and being located if one was lost. The persons with dementia carried the transmitter when they were alone outdoors and saw the value of it, in terms of their own as well as their spouse´ s feelings of safety and security. Safety and security aspects described by both persons with dementia and relatives have also been found in earlier studies on the use of tracking technologies in the care of persons with dementia [2,19,23,40]. The present study also identified the need for support and repeated practice using and handling the PPAP to promote a feeling of trust in the PPAP and in one´ s own ability to use it. The participants talked about the importance of the PPAP being easy to use, and this was expressed by the couples and observed in statements about the physical and functional design of the PPAP and about the abilities, knowledge and skills needed to be able to use the device (cf. [41]). The present results also revealed that, mostly for the spouses, trust in one´ s own abilities to use and handle the ICT and trust in the ICT itself were important for actually using the device. Although some persons with dementia felt no current need for the PPAP, describing themselves as ”too healthy”, all reported wanting the PPAP in the future. However, at the outset of the study, all couples described occasions when the person with dementia had gotten lost while he/she was alone outdoors. In view of this, it appears important that persons with dementia and their spouses receive adequate information and be given the opportunity to discuss different types of ICT with healthcare personnel. Such discussions with the couple, relatively early in the disease process, could facilitate joint decision-making regarding both current and future use of ICT in daily life (cf. [22]). In additional it is important that the technology being developed based on the needs and experiences of the person with dementia and their relatives and that such need-based development should go hand in hand with collaboration between the research community and private enterprise.

The present study showed that the couples´ perceptions of the PPAP increasing their safety overshadowed any risk of violating personal integrity. None of the couples in the present study spontaneously raised the ethical issue of whether use of the PPAP was intrusive, and the implementation of a hidden zone was not a desired feature. On the contrary, not being seen was considered a source of insecurity. These findings are in contrast to the ethical debate concerning tracking technology, which has focused on the risk of offending and violating the integrity of persons with dementia [24,30-32]. One explanation for this discrepancy may be that the PPAP used in the present study allowed only the spouses to monitor the persons with dementia on their outdoor walks. Several studies have reported that implementation of ICT in the care of persons with dementia needs to be preceded by extensive ethical discussions [11,32,42]. Welsh and colleagues [30] reported that some ICT (e.g., tracking technology) devices used in dementia care were perceived by healthcare staff as violating human dignity and freedom, given the use of similar technology in criminal surveillance. While this stance is understandable considering that all such devices are based on GPS, it could also be argued that it is unethical to deny persons with dementia and their relatives the opportunity to use a service, such as the PPAP, that could give them independence and security in daily life. It is also important that the tracking technology be adapted to the individual and used as an aid to living a daily life that is as fulfilling and active as possible despite the presence of a dementia disease. Finally, gender differences in testing and checking one’s own abilities as well as the PPAP were described and revealed in the present study. Further studies are needed to investigate these differences.

### Methodological considerations

The main strength of the present study was the use of repeated participant observations and informal conversations, an approach that allowed us to follow the couples and the development of their use of the PPAP over time [34] and can thereby be seen as appropriate. A further strength was that the observations and conversations were performed in the couples´ own homes and that the persons with dementia participated actively as informants. Despite the emotional upheaval of describing and reflecting on their situation, all of the participants (both persons with dementia and their spouses) appreciated being given an opportunity to speak with someone who showed an interest in hearing their stories. The observers´ knowledge and experience of caring for persons with dementia and their relatives were considered a prerequisite for admission to the home, for creating a trusting environment in which to carry out the observations and conversations, and for being given the opportunity and permission to return (on the next occasion) (cf. [35]).

Credibility [37,43] was achieved by the first and last authors discussing the steps used in the process of analyzing the observation and conversation occasions. All authors were engaged in a critical discussion of the analysis at all levels, from code to category. To further strengthen the credibility, excerpts from the interviews were included in the results. To increase the dependability of the study, the data collection and analysis process were continually discussed in the research team. The findings may be transferable to groups of people with other cognitive disabilities, although decisions about transferability must be made by the readers [34,37,43]. A limitation that should be taken into account is that data collection was carried out during a time of year when outdoor activity is limited, due to snow and low temperatures. This might have influenced the frequency of the couples´ use of the PPAP. Moreover, future research is needed to explore the experiences of persons with dementia in later stages of the disease. Persons with severe dementia may experience deterioration of their verbal communication skills, which is why a longitudinal observation study should be considered.

## Conclusions

The passive positioning alarm was perceived as providing valuable support for both persons with dementia and their spouses. Achieving successful implementation of the PPAP in the daily life of persons with dementia and their spouses requires a service package that provides an overall solution promoting the usability and value of the PPAP. The present study contributes to our understanding of what prerequisites need to be in place and what barriers need to be dealt with before successful implementation can occur. Due to the physical and functional design of the device, persons with dementia and their relatives must receive individual support that is based on their individual abilities, knowledge and skills. Such support and feedback at “the right level” should enable them to feel trust in themselves and in their use of the PPAP.

## Abbreviations

GPS: Global Positioning System; ICD: International Classification of Diseases; ICT: Information and Communication Technology; PPAP: Passive Positioning Alarm Package; SMS: Short Message Service

## Competing interests

The authors declare that they have no competing interests.

## Authors’ contributions

All authors participated in the design and analysis of the study and the manuscript preparation. Data collection was performed by AO. All authors read and approved the final manuscript.

## Pre-publication history

The pre-publication history for this paper can be accessed here:

http://www.biomedcentral.com/1471-2318/13/11/prepub
